# Polyarticular Septic Arthritis Caused by *Haemophilus Influenzae* in an Asplenic Patient: A Case Report

**DOI:** 10.5811/cpcem.20761

**Published:** 2025-01-19

**Authors:** Roberto Desarden, Roya Caloia

**Affiliations:** Henry Ford Genesys Hospital, Department of Emergency Medicine, Grand Blanc, Michigan

**Keywords:** polyarticular septic arthritis, sepsis, Haemophilus influenzae

## Abstract

**Introduction:**

Prevalence of serious infections from *Haemophilus influenzae* has diminished over the last few decades because of immunizations against the most virulent serotype. However, over the last few years a handful of septic arthritis cases secondary to *H influenzae* have been documented. Most of the cases documented are in the pediatric and unimmunized population. This is a case of polyarticular septic arthritis in a 69-year-old male who presented with syncope and ankle pain.

**Case report:**

A 69-year-old male presented to the emergency department after a syncopal event at home and complaining of right ankle pain. He was tachycardic and tachypneic on presentation and had an erythematous painful right ankle and right elbow. Aspiration of both joints produced purulent aspirate that grew *H influenzae*. Antibiotics were started, and the patient was taken to the operating room for emergent joint lavage. The patient made a full recovery and was discharged home with a peripherally inserted central catheter line for continued intravenous (IV) antibiotics.

**Conclusion:**

Our case highlights an atypical presentation for a case of polyarticular septic arthritis caused by *H influenzae*. We were unable to rule out endocarditis as a source of the bacterial seeding, and the patient improved with IV antibiotics and surgical lavage of the affected joints.

## INTRODUCTION

*Haemophilus influenzae* is a Gram-negative coccobacillus that is notorious for causing serious infections such as meningitis, endocarditis, and epiglottitis.^1^ This bacteria has been identified with an encapsulated (typeable) and unencapsulated (untypeable) form.^2^ *Haemophilus influenzae* serotype b is known to be the most virulent. Vaccination against this strain has helped prevent life-threatening illness.^3^ Even with the increasing prevalence of non-typeable *H influenzae*, there have been occasional accounts of *H influenzae* causing polyarticular septic arthritis.^4^ In this report, we discuss the presentation of a 69-year-old male with polyarticular septic arthritis secondary to *H influenzae*.

## CASE REPORT

A 69-year-old male with a past medical history of hypertension, gastroesophageal reflux disease, and prior splenectomy presented to the emergency department (ED) via private vehicle for evaluation of syncope and right ankle pain. He stated that he was at home walking down the hallway when he started to feel lightheaded and had a syncopal event. He was down for a short amount of time since his wife was present during the event and helped him to his feet. He denied any head trauma but complained of right ankle pain, right elbow pain, myalgias, and rigors after the syncopal episode. The pain in his ankle and elbow was exacerbated with any movement. He believed he strained his joints during the syncopal event. Because of his rigors, he performed a home COVID-19 test, which was negative. He denied any fever, rhinorrhea, sore throat, cough, shortness of breath, chest pain, nausea, vomiting, diarrhea, recent travel, or any sick contacts.

**Image 1:**
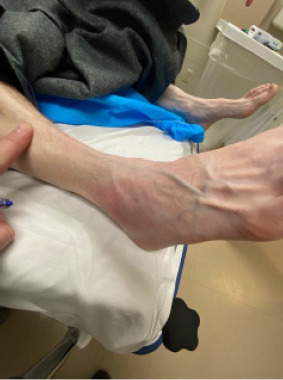
Erythematous right ankle of the patient.

The patient appeared uncomfortable but not in any distress. His temperature was 98.3° Fahrenheit; heart rate, 104 beats per minute (min); blood pressure, 111/71 millimeters of mercury; respiratory rate, 20 breaths per min; and oxygen saturation, 96% on room air. On exam, his right ankle was erythematous, warm to the touch, and edematous over the lateral malleolus (Image 1).

There was point tenderness over the lateral malleolus without any crepitus. Plantar flexion of the right foot significantly exacerbated the pain in the ankle. The right elbow had no overlying erythema or edema but was tender to palpation over the olecranon process. Extension of the elbow exacerbated the pain. Cardiopulmonary auscultation demonstrated regular tachycardia without any murmurs, wheezes, rhonchi, or rales. The remainder of the exam was normal.

**Image 2:**
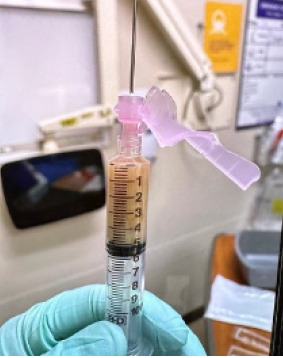
Ankle joint purulent aspirate.

The patient was given a 30 milliliters per kilogram bolus of normal saline, and blood cultures were obtained. Orthopedic surgery was consulted, and arthrocentesis of both joints was performed. Purulent material was aspirated from the right ankle and right elbow (Image 2).

**CPC-EM Capsule**

What do we already know about this clinical entity?

Haemophilus influenzae *septic polyarthritis cases are rare and have mostly been documented in pediatric and unimmunized patients*.

What makes this presentation of disease reportable?

*In this case the joint infection was caused by an uncommon organism with no confirmed source of infection in an immunized but asplenic patient*.

What is the major learning point?

*It’s important to consider transesophageal echocardiogram to rule out endocarditis if transthoracic echocardiogram is negative when there is high clinical suspicion*

How might this improve emergency medicine practice?

*We highlight the importance of the physical and differential while providing insight on testing considerations in an atypical presentation of an orthopedic emergency*.

Vancomycin and ceftriaxone were then started empirically to treat septic arthritis. A complete blood count showed a leukocytosis of 39.1×10^3^ per mm^3^ (reference range 4.5–11.0 ×10^3^/mm^3^) and absolute neutrophil count of 34.6 ×10^3^/mm^3^ (1.0–8.0 × 10^3^/mm^3^). Basic metabolic panel showed an anion gap of 15 millimoles per liter (mmol/L) (8–16 mmol/L); bicarbonate, 24 mmol/L (20–30 mmol/L); creatinine, 1.8 milligrams per deciliter (mg/dL) (0.61–1.24 mg/dL); and blood urea nitrogen, 45 mg/dL (8–26 mg/dL). Lactic acid was 3.2 mmol/L (0.50–2.20 mmol/L). Erythrocyte sedimentation rate was 65 mm per hour (mm/hr) (2–10 mm/hr) with C-reactive protein, 375 mg/L (0.00–5.00 mg/L). Cell count of the right elbow aspirate showed 205,300 nucleated cells/microliter (NUC/μL) (0–200 NUC/μL). Cell count of the right ankle was not performed as the sample was too turbid and viscous. Gram stain of the joint aspirate revealed Gram-negative rods in the aerobic and anaerobic bottle. Plain film radiography of the right ankle and elbow showed soft-tissue swelling, without evidence of gas or fracture.

The patient was taken to the operating room where arthrotomy and irrigation of both joints was performed. A large amount of purulent material was removed during the procedure, and blunt debridement of the joints was performed. Blood cultures and both joint aspirate samples grew *H influenzae* that was sensitive to beta-lactam antibiotics. Unfortunately, our institution was unable to determine whether the isolate was encapsulated or unencapsulated. Transthoracic echocardiogram (TTE) was negative for any valvular vegetations. He was discharged on hospital day six with a peripherally inserted central line in place for outpatient antibiotic treatment. The patient’s childhood immunizations were up to date, and he was scheduled for follow-up to see an infectious disease specialist to update his vaccines against encapsulated organisms.

## DISCUSSION

Septic arthritis is usually caused by hematogenous spread or by traumatic inoculation.^5^ Treatment involves arthrotomy and irrigation of the affected large joint spaces in combination with the appropriate antibiotic regimen.^6^ *Haemophilus influenzae* septic arthritis can be treated with 14–21 days of intravenous (IV) ceftriaxone.^7^ We believe the route of entry for the bacteria in our patient was most likely oral, given that *H influenzae* is primarily an upper respiratory organism in humans.^8^ The patient’s history of a splenectomy likely predisposed him to a more serious infection. A TTE was negative for evidence of endocarditis in this case, but it is not the gold standard as its sensitivity is only 40–63%. In comparison, a transesophageal echocardiogram (TEE) has a sensitivity of 90–100%.^9^ No justification was made to explain why a TEE wasn’t obtained, but it would have been an important diagnostic test to rule out endocarditis as the source of the patient’s bacteremia. It is possible that the reading cardiologist may have felt comfortable ruling out endocarditis with the TTE, but that would be speculation. A TEE is a more comprehensive, time- and personnel-intensive test and what we would recommend being done as a next step. Unfortunately, in our patient we cannot definitively say we ruled out endocarditis and, therefore, he was treated presumptively for endocarditis with four weeks of IV ceftriaxone.

## CONCLUSION

This case highlights an atypical presentation of polyarticular septic arthritis caused by an uncommon organism in an uncommon demographic. The patient initially believed his elbow and ankle pain were secondary to a strain from a syncopal episode. In addition to highlighting the importance of keeping polyarticular septic arthritis in the differential, this case demonstrates the need to not anchor on presumptive diagnoses provided by patients and to perform a thorough history and physical exam to come up with a differential diagnosis. Polyarticular septic arthritis was diagnosed, and our patient made a full recovery after joint lavage and antibiotic therapy.
